# Supplementary material for: Sequenced Combinations of Cisplatin and Selected Phytochemicals towards Overcoming Drug Resistance in Ovarian Tumour Models
Source: Int J Mol Sci. 2020 Oct 12;21(20):7500. doi: 10.3390/ijms21207500 (PMC7589098; doi:10.3390/ijms21207500)
Supplement: Supplementary file 1 [file ijms-21-07500-s001.pdf]

Supplementary Materials:

Table 1. Characteristics of proteins that have undergone differential expressions in A2780 and A2780^{cisR} cell lines following treatment with selected drug combinations using A2780 cell line as reference.

Mach ID	Protein ID	Full name	Mascot search results	Location	References
3	CYPA<br>P62937	Cyclophilin A	Mass: 18001<br>Mascot score: 252<br>Coverage: 53%<br>*pI:* 7.68<br>MS:15<br>MSMS:3	Cytoplasm	[15]<br>http://www.matrixscience.com
4	EIF5A1<br>P63241	Eukaryotic initiation factor 5A isoform 1	Mass: 16821<br>Mascot score:168<br>Coverage: 51%<br>*pI*:5.08<br>MS:7<br>MSMS:2	Cytoplasm<br>Nucleus.<br>Endoplasmic reticulum membrane	[15]<br>http://www.matrixscience.com
5	p18<br>P23528	18 kDa phosphoprotein	Mass: 18491<br>Mascot score: 137<br>Coverage: 42%<br>*pI*: 8.22<br>MS:14<br>MSMS:2	Nucleus matrix.<br>Cytoplasm, cytoskeleton.<br>cell projection	[15]<br>http://www.matrixscience.com
7	Op18<br>P16949	Oncoprotein 18	Mass: 17292<br>Mascot score: 114<br>Coverage: 53%<br>*pI*: 5.76<br>MS:11<br>MSMS:2	Cytoplasm,<br>cytoskeleton	[15]<br>http://www.matrixscience.com
8	PRDX1<br>Q06830	Peroxiredoxin-1	Mass: 22096<br>Mascot score: 400<br>Coverage: 59%<br>*pI*: 8.27<br>MS:20<br>MSMS:6	Cytoplasm	[15]<br>http://www.matrixscience.com
11	PGAM1<br>P18669	Phosphoglycerate mutase 1	Mass: 28786<br>Mascot score: 318<br>Coverage: 64%<br>*pI*: 6.67<br>MS:17<br>MSMS:3		[15]<br>http://www.matrixscience.com
13	1433Z<br>P63104	14-3-3 protein zeta/delta	Mass: 27728<br>Mascot score: 216<br>Coverage: 61%<br>*pI*: 4.73<br>MS:22<br>MSMS:4	Cytoplasm	[15]<br>http://www.matrixscience.com
15	hnRNPA1<br>P09651	Heterogeneous nuclear ribonucleoprotein A1	Mass: 38723<br>Mascot score: 325<br>Coverage: 46%<br>*pI*: 9.17<br>MS:22<br>MSMS:5	Nucleus.<br>Cytoplasm	[15]<br>http://www.matrixscience.com
16	hnRNPA2/B1<br>P22626	Heterogeneous nuclear ribonucleoproteins A2/B1	Mass: 37407<br>Mascot score: 267<br>Coverage: 49%<br>*pI*: 8.97<br>MS:19<br>MSMS:4	Nucleus,<br>nucleoplasm.<br>Cytoplasm	[15]<br>http://www.matrixscience.com
17	ENOA	Alpha-enolase	Mass: 37407<br>Mascot score: 331<br>Coverage 44%	Cytoplasm. Cell membrane.	[15]<br>http://www.matrixscience.com

			pI: 7.01<br>MS:23<br>MSMS:5		
19	LDHB<br>P07195	L-lactate dehydrogenase B chain;	Mass: 36615<br>Mascot score: 81<br>Coverage: 46%<br>*pI*: 5.71<br>MS:24<br>MSMS:6	Cytoplasm	[15]<br>http://www.matrixscience.com
25	EFTU<br>P49411	Elongation factor Tu, mitochondrial	Mass: 49510<br>Mascot score: 499<br>Coverage: 49%<br>*pI*: 7.26<br>MS:22<br>MSMS:7	Mitochondrion	[15]<br>http://www.matrixscience.com
27	EF1G<br>P26641	Elongation factor 1-gamma	Mass: 50087<br>Mascot score: 389<br>Coverage: 42%<br>*pI: 6.25*<br>MS:23<br>MSMS:5		[15]<br>http://www.matrixscience.com
31	ENOA<br>P06733	Alpha-enolase	Mass: 47139<br>Mascot score: 331<br>Coverage: 44%<br>*pI*: 7.01<br>MS:23<br>MSMS:5	Cytoplasm. Cell membrane	[15]<br>http://www.matrixscience.com
32	TCPB<br>P78371	T-complex protein 1 subunit beta	Mass: 57452<br>Mascot score: 91<br>Coverage: 39%<br>*pI*: 6.01<br>MS: 30<br>MSMS: 0	Cytoplasm	[15]<br>http://www.matrixscience.com
33	SERA<br>O43175	D-3-phosphoglycerate dehydrogenase	Mass: 56614<br>Mascot score: 213<br>Coverage: 25%<br>*pI*: 6.29<br>MS: 15<br>MSMS: 4	Cytosol	[15]<br>http://www.matrixscience.com
35	ERp57<br>P30101	Endoplasmic reticulum resident protein 57	Mass: 56747<br>Mascot score: 521<br>Coverage: 48%<br>*pI*: 5.98<br>MS: 6<br>MSMS: 31	Endoplasmic reticulum	[15]<br>http://www.matrixscience.com
36	VIME<br>P08670	Vimentin	Mass: 53619<br>Mascot score: 609<br>Coverage: 75%<br>*pI*: 5.06<br>MS: 51<br>MSMS: 4	Cytoplasm	[15]<br>http://www.matrixscience.com
39	TCPH<br>Q99832	T-complex protein 1 subunit eta	Mass: 59329<br>Mascot score: 84<br>Coverage: 25%<br>*pI: 7.55*<br>MS: 19<br>MSMS: 2	Cytoplasm	[15]<br>http://www.matrixscience.com
45	HSP7C<br>P11142	Heat shock cognate 71 kDa protein	Mass: 70854<br>Mascot score: 705<br>Coverage: 62%<br>*pI: 5.37*<br>MS: 45<br>MSMS: 6	Cytoplasm	[15]<br>http://www.matrixscience.com
46	Mortalin<br>P38646	Stress-70 protein	Mass: 73635<br>Mascot score: 189<br>Coverage: 17%	Mitochondrion	[15]<br>http://www.matrixscience.com

			pI: 5.87<br>MS: 10<br>MSMS: 5		
47	BIP<br>P11021	Immunoglobulin heavy chain-binding	Mass: 72288<br>Mascot score: 345<br>Coverage: 46%<br>*pI: 5.07*<br>MS: 33<br>MSMS:6	Endoplasmic reticulum lumen	[15]<br>http://www.matrixscience.com
51	HSP90B<br>P08238	Heat shock protein HSP 90-beta	Mass: 83212<br>Mascot score: 623<br>Coverage: 40%<br>*pI: 4.97*<br>MS: 37<br>MSMS:7		[15]<br>http://www.matrixscience.com
52	EF2<br>P13639	Elongation factor 2	Mass: 95277<br>Mascot score: 431<br>Coverage: 32%<br>*pI*: 6.41<br>MS: 47<br>MSMS:7		[15]<br>http://www.matrixscience.com
54	GRP94<br>P14625	94 kDa glucose-regulated protein	Mass: 92411<br>Mascot score: 99<br>Coverage: 22%<br>*pI: 4.76*<br>MS: 18<br>MSMS:2	Endoplasmic reticulum lumen	[15]<br>http://www.matrixscience.com
55	IMMT<br>Q16891	Mitochondrial inner membrane protein	Mass: 83626<br>Mascot score: 136<br>Coverage: 29%<br>*pI: 6.08*<br>MS: 23<br>MSMS:1	Mitochondrion inner membrane	[15]<br>http://www.matrixscience.com
63	NM23<br>P15531	Metastasis inhibition factor nm23	Mass: 17138<br>Mascot score: 405<br>Coverage: 60%<br>*pI*: 5.83<br>MS: 12<br>MSMS:6	Cytoplasm	[15]<br>http://www.matrixscience.com
66	PRDX6<br>P30041	Peroxiredoxin-6	Mass: 25019<br>Mascot score: 342<br>Coverage: 51%<br>*pI*: 6.00<br>MS: 15<br>MSMS:5	Cytoplasm	[15]<br>http://www.matrixscience.com
69	PSA3<br>P25788	Proteasome subunit alpha type-3	Mass: 28415<br>Mascot score: 125<br>Coverage: 50%<br>*pI: 5.19*<br>MS: 15<br>MSMS: 3	Cytoplasm	[15]<br>http://www.matrixscience.com
76	hnRNPA2/B1<br>P22626	Heterogeneous nuclear ribonucleoproteins A2/B1	Mass: 37407<br>Mascot score: 220<br>Coverage: 55%<br>*pI*: 8.97<br>MS: 20<br>MSMS: 2	Nucleus, nucleoplasm	[15]<br>http://www.matrixscience.com
88	Hop<br>P31948	Hsc70/Hsp90-organizing protein	Mass: 62599<br>Mascot score: 375<br>Coverage: 52%<br>*pI: 6.40*<br>MS: 45<br>MSMS: 6	Cytoplasm (By similarity).	[15]<br>http://www.matrixscience.com
89	MCM7<br>P33993	DNA replication licensing factor MCM7	Mass: 81257<br>Mascot score: 214<br>Coverage: 54%	Nucleus (By similarity)	[15]<br>http://www.matrixscience.com

			pI: 6.08<br>MS: 40<br>MSMS:3		
94	GBLP<br>P63244	Guanine nucleotide-binding protein subunit beta-2-like 1	Mass: 35055<br>Mascot score: 189<br>Coverage: 37%<br>*PI: 7.60*<br>MS: 16<br>MSMS:2	Cell membrane; Peripheral membrane protein.	[15]<br>http://www.matrixscience.com
95	PSAT<br>Q9Y617	Phosphohydroxythreonine aminotransferase	Mass: 40397<br>Mascot score: 437<br>Coverage: 41%<br>*pI: 7.56*<br>MS: 21<br>MSMS:7	(UniPortKB 2014) (http://www.matrixscience.com)	[15]<br>http://www.matrixscience.com
96	VIME<br>P08670	Vimentin	Mass: 53619<br>Mascot score: 452<br>Coverage: 71%<br>*pI*: 5.06<br>MS: 48<br>MSMS:7	Cytoplasm.	[15]<br>http://www.matrixscience.com
97	ATPA<br>P25705	ATP synthase subunit alpha, mitochondrial	Mass: 59714<br>Mascot score: 363<br>Coverage: 56%<br>*pI: 9.16*<br>MS: 32<br>MSMS:5	Mitochondrion inner membrane	[15]<br>http://www.matrixscience.com
102	RUVB1<br>Q9Y265	RuvB-like 1	Mass: 50196<br>Mascot score: 106<br>Coverage: 24%<br>*pI: 6.02*<br>MS: 17<br>MSMS:4	Nucleus matrix	[15]<br>http://www.matrixscience.com
105	TCPA<br>P17987	T-complex protein 1 subunit alpha	Mass: 60306<br>Mascot score: 167<br>Coverage: 43%<br>*pI: 5.80*<br>MS: 21<br>MSMS:3	Cytoplasm	[15]<br>http://www.matrixscience.com
108	CAH2<br>P00918	Carbonic anhydrase 2	Mass: 29228<br>Mascot score: 159<br>Coverage: 27%<br>*pI: 6.87*<br>MS: 7<br>MSMS:3	Cytoplasm	[15]<br>http://www.matrixscience.com
116	PRS7<br>P35998	26S protease regulatory subunit 7	Mass: 48603<br>Mascot score: 98<br>Coverage: 45%<br>*pI: 5.71*<br>MS: 20<br>MSMS: 1	Cytoplasm	[15]<br>http://www.matrixscience.com

* Accession number, protein ID, names and Mass, MSMS, coverage and protein score were obtained from APAF (http://www.proteome.org.au/); *Theoretical isoelectric point (pI), subcellular location mass spectrum and matched peptides were obtained from Mascot database (http://www.matrixscience.com), SwissProt database), (http://www.uniprot.org/) .

[15]; *Protein scores from Mascot database (http://www.matrixscience.com) through APAF (http://www.proteome.org.au/).
